# Hand-assisted laparoscopic surgery for Bochdalek hernia in an adult patient with a history of laparotomy: A case report

**DOI:** 10.1016/j.ijscr.2019.06.022

**Published:** 2019-06-17

**Authors:** Shogo Fukutomi, Shoichirou Arai, Masahiro Fujisaki, Kazuya Naritomi, Masahiro Kawabata, Masae Mano

**Affiliations:** Department of Surgery, Saiseikai Futsukaichi Hospital, 3-13-1, Yumachi, Chikushino-shi, Fukuoka, Japan

**Keywords:** Bochdalek hernia, Hand-assisted laparoscopic surgery, Abdominal incisional hernia, Case report

## Abstract

**Introduction:**

Bochdalek hernia (BH) is a congenital diaphragmatic hernia that generally occurs in infants and is rarely seen in adults. Surgical repair of BH is recommended, but the approach for repairing BH should be selected carefully in individual cases. It is well known that hand-assisted laparoscopic surgery (HALS) has the advantage of preserving tactile sensation compared with standard laparoscopic surgery. We describe an adult patient with a history of abdominal incisional hernia who developed BH that was treated safely by HALS.

**Presentation of case:**

An 87-year-old woman was admitted to our hospital with nausea. She had a history of right hemicolectomy and repair of an abdominal incisional hernia using mesh at 5 years after hemicolectomy. Chest and abdominal computed tomography revealed herniation of the gastric corpus through the left posterior diaphragm. BH was diagnosed and hernia repair by HALS was selected as the approach because dense adhesions were expected in the abdominal cavity. The operation was performed safely and her postoperative course was uneventful.

**Conclusion:**

HALS was useful, especially when removing adhesions around the hernial orifice. HALS is a feasible approach for BH and should be considered as one of the options in patients with a history of previous abdominal surgery.

## Highlights

• Bochdalek hernia (BH) is a congenital diaphragmatic hernia and rarely seen in adults.
• Surgical approach is required for BH, but the optimal method is still controversial.
• Hand assisted laparoscopic surgery (HALS) allowed us to clear the adhesions easily.
• HALS should be considered as one of the options for adult BH in selected patients.

## 1 Introduction

Bochdalek hernia (BH) is a type of congenital diaphragmatic hernia that mainly occurs in infants due to failure of diaphragmatic closure. Therefore, BH is relatively rare in adults and has been reported to account for between 0.17–6% of adult diaphragmatic hernias [1,2]. Surgical reduction of the abdominal organs and closure of the defect is required for BH, but the optimal method is still controversial [1] and should be selected carefully according to the patient’s condition. Although a number of recent reports have described the efficacy of laparoscopic repair for BH in adults [1,[3], [4], [5]], there have been no reports about the use of hand-assisted laparoscopic surgery (HALS) for this purpose. Here we report safe treatment of BH by HALS in an adult with a history of abdominal incisional hernia. This case report has been reported in line with the SCARE criteria [6].

## 2 Case report

**Fig. 1:**
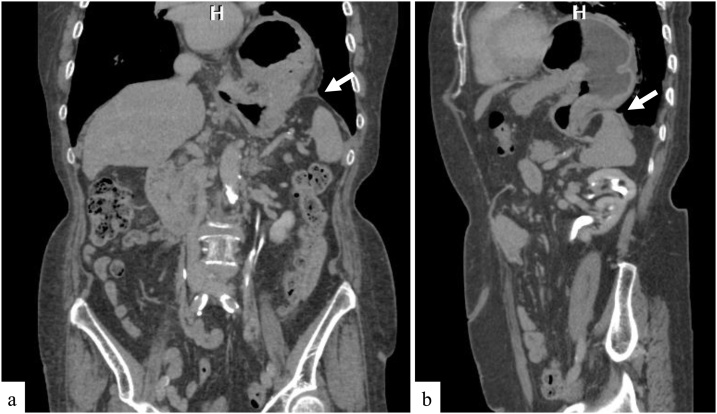
Preoperative images of the Bochdalek hernia. CT reveals herniation of the gastric corpus through the left posterior diaphragm (white arrow) in a coronal view (a) and a sagittal view (b).

**Fig. 2:**
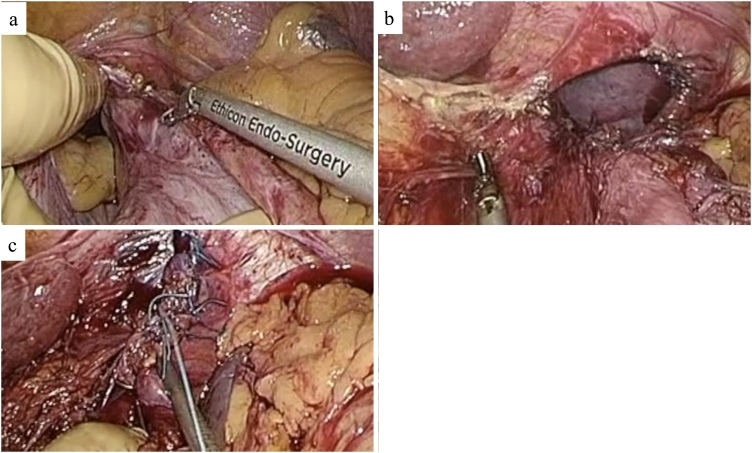
HALS for repair of the Bochdalek hernia. a. Adhesions around the hernial orifice were carefully removed by HALS. b. A 5 × 3 cm hernial orifice with a sac was observed. c. The defect was repaired with interrupted nonabsorbable sutures.

An 86-year-old woman who had been living with her elderly husband was admitted to our hospital with the complaints of nausea, anorexia, and epigastric discomfort. When she was 73 years old, the patient had undergone laparotomy and right hemicolectomy for resection of colon cancer. Five years after hemicolectomy, she had received hernia repair surgery using mesh for an abdominal incisional hernia. There was no history of abdominal or thoracic trauma. Laboratory tests were all within the normal range. Her electrocardiogram showed no ischemic changes. Although chest X-ray revealed an abnormal gas-filled mass in the left thoracic cavity, the patient had no symptom of dyspnea. Contrast-enhanced computed tomography (CT) scan confirmed herniation of the gastric corpus through the left posterior part of the diaphragm (Fig. 1). We diagnosed adult Bochdalek hernia and planned its surgical treatment. As mesh had been placed under the previous upper abdominal midline incision more than 10 years earlier, dense adhesions between the mesh and abdominal tissues were expected. Therefore, we decided to perform diaphragmatic hernia repair by HALS, considering the patient’s safety. After induction of general anesthesia, the patient was placed in the supine position with her legs apart. The previous midline incision was opened carefully to insert a LAP DISC^®^ (Hakko, Nagano, Japan) for a hand port. Dense adhesions, which had to be divided, were found between the mesh used to repair her incisional hernia and loops of the small intestine. The disk for HALS was placed after complete removal of the mesh. Subsequently, a 12 mm trocar was inserted into the inferior umbilical region for the laparoscope. A 5 mm trocar was also inserted into the left upper abdominal region. Herniation of the gastric corpus into the left thoracic cavity through a hernial orifice in the left posterior diaphragm was confirmed. The gastric corpus could not be pulled back into the abdominal cavity because of adhesions around the hernial orifice. After these adhesions were carefully removed by HALS using Harmonic ACE shears (Ethicon, NJ, USA) (Fig. 2a), the stomach was completely freed and could be returned to the abdominal cavity. A 5 × 3 cm hernial defect with sac was observed (Fig. 2b). We decided to perform simple closure of the defect without mesh reinforcement because the rim of the hernial orifice was relatively strong. Accordingly, the defect was repaired with interrupted nonabsorbable sutures (2-0 Nesporen; Alfresa Pharma Corporation, Osaka, Japan) using a 5 mm port on the left upper abdomen and the surgeon’s left hand via the hand port (Fig. 2c). The midline incision for the hand port was closed without mesh reinforcement. A drain tube was placed under the left hemidiaphragm. The operating time was 244 min and there was no significant bleeding. Her postoperative course was uneventful. The patient was discharged on postoperative day 20. There was no evidence of recurrence at 1-year follow-up.

## 3 Discussion

BH is the most common type of congenital diaphragmatic hernia and it was first described by Bochdalek in 1848. It is thought to occur due to incomplete formation of the posterolateral diaphragm during weeks 8–10 of gestation [2].

Diagnosis of BH in adults is often difficult because the symptoms are atypical, including nausea, cough, dyspnea, chest pain, and abdominal pain [1,2]. In fact, the misdiagnosis rate was reported to be as high as 38% [3,7]. However, accurate diagnosis of BH is extremely important because delay in making the diagnosis often leads to fatal complications, such as strangulated ileus with necrosis of the small intestine [3,7,8].

The most frequent etiology of acquired diaphragmatic hernia in adults is secondary to trauma [9]. It was reported approximately 5% of the patients with abdominal trauma had a diaphragmatic injury at the operation. Almost all diaphragmatic injuries were caused by blunt trauma of the chest and abdomen (75%), and rarely by stabbing (25%) [10]. On the other hand, the cause of BH in adults remains unknown. It has been suggested that elevation of the intra-abdominal pressure by strenuous exercise, pregnancy, or trauma may be associated with a risk of BH. Our patient had a history of surgery for abdominal incisional hernia. Thus, the increase of intra-abdominal pressure after abdominal hernia repair might have been one of the precipitating factors for diaphragmatic hernia in this case.

The surgical treatment of diaphragmatic hernia is generally performed by laparotomy, thoracotomy, laparoscopy, thoracoscopy, or a combined thoracoabdominal approach [1,4,7]. It was reported that laparotomy was widely used in 184 patients who underwent surgical intervention from 1955 to 2015 [1]. Laparotomy was especially preferred in emergency cases, but the approach for repair of BH depends on various factors in individual patients, such as the degree of emergency, the size of the defect, and the presence of complications. Moreover, it sometimes depends on the surgeon’s experience [1]. Agrusa et al. reported the efficacy of the re-laparoscopic approach for the management of postoperative complications in order to avoid further complications [11]. Although the laparoscopic approach is the best maneuver with regards to minimal invasiveness, it has the possibility of requiring a long operative time in some cases. In the current patient, dense adhesions were expected because there was a history of previous surgical intervention. We considered that laparoscopic adhesiolysis would take a long time and was unsuitable for this patient, but minimally invasive surgery was still required because the patient was elderly. Therefore, the decision was made to perform HALS via the old incision after removing the mesh that had been used to repair her abdominal hernia. This technique provided better tactile sensation and allowed us to easily clear the adhesions around the hernial orifice through direct manual maneuvers. HALS has certain advantages, especially in the situation where the contents of the hernial sac need to be extracted and dissected free of the hernial orifice. In addition, the direct use of the surgeon’s left hand via the disk facilitates the sutures for the hernial orifice. The surgical outcome in our patient was comparable to that in previously reported patients treated by laparoscopy [1,[3], [4], [5]]. HALS is a hybrid procedure that allows the surgeon to insert the non-dominant hand into the abdomen under laparoscopic guidance. This approach provides the tactile experience of open surgery together with the good visualization and low invasiveness characteristic of laparoscopic surgery [[12], [13], [14]]. HALS can be considered as an alternative to laparoscopic surgery for BH repair in selected patients.

## 4 Conclusion

We performed safely HALS to repair BH in an adult. HALS is a feasible approach for BH and should be considered as one of the treatment options in patients with a history of previous abdominal surgery.

## Conflicts of interest

The authors declare that they have no competing interests.

## Sources of funding

This research did not receive any specific grant from funding agencies in the public, commercial, or not-for-profit sectors.

## Ethical approval

Institutional review board approval was exempt from our institution because all data were collected from clinical records and imaging systems for routine preoperative planning and follow up.

## Consent

Informed consent was obtained from the patient for publication of this case report and any accompanying images.

## Author’s contribution

SF, SA, MF, and KN are surgeons who operated on the patients. SF wrote the initial draft of the manuscript. MK and MM supervised the preparation of this case report. All authors have read and approved the final manuscript.

## Registration of research studies

This paper reports just the record of patient treatment. This is not a paper about research work involving human participants.

## Guarantor

Masahirio Kawabata.

## Provenance and peer review

Not commissioned, externally peer-reviewed.
